# Developing a home-based pulmonary rehabilitation programme for patients with chronic respiratory diseases in Malaysia: A mixed-method feasibility study

**DOI:** 10.7189/jogh.13.04099

**Published:** 2023-10-27

**Authors:** Soo Chin Chan, Julia Patrick Engksan, Jayakayatri Jeevajothi Nathan, Jaspreet Kaur Sekhon, Norita Hussein, Anwar Suhaimi, Nik Sherina Hanafi, Yong Kek Pang, Saari Mohamad Yatim, G M Monsur Habib, Hilary Pinnock, Ee Ming Khoo, RESPIRE Collaboration

**Affiliations:** 1 Department of Rehabilitation Medicine, Faculty of Medicine, Universiti Malaya, Kuala Lumpur, Malaysia; 2 Department of Primary Care Medicine, Faculty of Medicine, Universiti Malaya, Kuala Lumpur, Malaysia; 3 Department of Medicine, Faculty of Medicine, Universiti Malaya, Kuala Lumpur, Malaysia.; 4 Department of Rehabilitation Medicine, Serdang Hospital, Selangor, Malaysia; 5 Bangladesh Primary Care Respiratory Society, Khulna, Bangladesh; 6 NIHR Global Health Research Unit on Respiratory Health (RESPIRE), Usher Institute, University of Edinburgh, Edinburgh, UK

## Abstract

**Background:**

The COVID-19 pandemic has underscored the importance of remote healthcare and home-based interventions, including pulmonary rehabilitation, for patients with chronic respiratory diseases (CRDs). It has also heightened the vulnerability of individuals with underlying respiratory conditions to severe illness from COVID-19, necessitating exploration and assessment of the feasibility of delivering home – pulmonary rehabilitation (home-PR) programmes for CRD management in Malaysia and other countries. Home-based programmes offer a safer alternative to in-person rehabilitation during outbreaks like COVID-19 and can serve as a valuable resource for patients who may be hesitant to visit healthcare facilities during such times. We aimed to assess the feasibility of delivering a home-PR programme for patients with CRDs in Malaysia.

**Methods:**

We recruited patients with CRDs from two hospitals in Klang Valley, Malaysia to a home-PR programme. Following centre-based assessment, patients performed the exercises at home (five sessions/week for eight weeks (total 40 sessions)). We monitored the patients via weekly telephone calls and asked about adherence to the programme. We measured functional exercise capacity (6-Minutes Walking Test (6MWT) and Health-Related Quality-of-Life (HRQoL) (COPD Assessment Test (CAT)) at baseline and post-PR at nine weeks. We conducted semi-structured interviews with 12 purposively sampled participants to explore views and feedback on the home-PR programme. The interviews were audio recorded, transcribed verbatim, and analysed thematically.

**Results:**

We included 30 participants; two withdrew due to hospitalisation. Although 28 (93%) adhered to the full programme, only 11 (37%) attended the post-PR assessment because COVID-19 movement restrictions in Malaysia at that time prevented attendance at the centre. Four themes emerged from the qualitative analysis: involvement of family and caregivers, barriers to home-PR programme, interactions with peers and health care professionals, and programme enhancement.

**Conclusion:**

Despite the COVID-19 pandemic, the home-PR programme proved feasible for remote delivery, although centre-based post-PR assessments were not possible. Family involvement played an important role in the home-PR programme. The delivery of this programme can be further improved to maximise the benefit for patients.

Chronic respiratory diseases (CRDs), which include chronic obstructive pulmonary disease (COPD), asthma, bronchiectasis, interstitial lung disease (ILD), post-tuberculosis (post-TB), and lung cancer survivors, contribute to 7.4% of the global disease burden [1]. People with CRDs experience disabling symptoms such as breathlessness, reduced exercise intolerance, cough, and poor quality of life. Pulmonary rehabilitation (PR), a comprehensive intervention that includes a personalised exercise programme and non-exercise/educational components, was found to be beneficial regardless of age, gender, severity of airflow limitation, or setting (hospital, outpatient clinic, or home) [2]. For COPD, attending a PR programme improves exercise capacity and health- related quality-of-life (HRQoL), reduces symptoms, exacerbations, and mortality [3]. It is widely recommended for people with other CRDs presenting with similar signs and symptoms [4]. In many diseases, low exercise capacity is associated with worse survival [5]; improving it through PR can potentially improve long-term disease outcomes [6], but access to PR is scarce or non-existent in many low- and middle-income countries (LMICs).

In Malaysia, PR is recommended in the management of COPD [7], but the limited access to the programme has been further compromised during the coronavirus 2019 (COVID-19) pandemic by movement restrictions and social distancing measures. Yet despite lockdown periods, patients with CRDs need to have continued access to PR [8], as strong evidence suggests that home-PR programmes provide outcomes comparable to centre-based PR [9-12]. Compared to usual care, our recent meta-analysis showed that home-PR programme significantly improved exercise capacity (standardised mean difference (SMD) = 0.88; 95% confidence interval (CI) = 0.32 to 1.44, *P* = 0.002) and HRQoL (SMD = -0.62; 95% CI = -0.88 to -0.36, *P* = 0.001) [13], enabling people with CRDs to continue receiving rehabilitation even if they are unable to attend the centre-based PR. In this study, we worked with local rehabilitation experts to adapt a PR programme to home-based delivery using minimal resources [14,15]. As home-PR programme is novel in Malaysia, we aimed to assess the feasibility of delivering the programme for patients with CRDs and explore the perceptions of patient and professional stakeholders. The findings of this study will help us to refine current PR programmes and develop implementation strategies in Malaysia.

## Methods

### Study design and setting

Aligned with the developmental phases of the Medical Research Council Framework for the development and evaluation of complex interventions, we conducted a mixed-methods qualitative and quantitative feasibility study between March and July 2021 in two hospitals in the Klang Valley, Malaysia; a teaching hospital that provides physiotherapist-led supervised exercise for patients with CRDs, and a Ministry of Health’s specialist hospital with an established eight-week centre-based PR programme.

### Study population

We recruited 30 participants between 18 March and 23 April 2021 through convenience sampling based on pre-defined inclusion/exclusion criteria. We did perform randomisation, as we conducted the study during the COVID-19 pandemic while movement restriction orders were in place, making it impossible to have another arm doing a hospital-based PR.

We included adult patients over 18 years old with CRDs (comprising COPD, post-TB, lung fibrosis, bronchiectasis, and ILD) who had previously received pulmonary physiotherapy. The patients had to understand Malay and English and agree to be monitored via phone call. We excluded patients with any of the following conditions: non-respiratory causes of symptoms, such as breathlessness due to heart failure or anaemia; co-morbidities that are contraindications to PR, such as unstable angina, aortic aneurysm, recent myocardial infarction, acute infection including pneumonia, active pulmonary tuberculosis; significant cognitive impairment preventing participation; inability to participate in exercise due to severe arthritis or paralysis; and inability to provide written consent. For the qualitative component, we invited four patients with CRDs, four family members of patient with CRDs, and four healthcare personnel (HCPs) (one medical officer, two physiotherapists and a medical assistant) from both hospitals to participate. We conducted the qualitative interviews with the aid of a topic guide (Text S1 in the **Online Supplementary Document**).

### The home-PR programme

The eight-week home-PR programme comprises nine components: exercise training (upper and lower limb stretching and strengthening), endurance (walking, cycling or other endurance activities), education (disease knowledge and medication), pharmacological treatment, self-management skills (e.g. inhaler technique and airway clearance), coping strategies, breathing techniques, and nutrition. In this study, each patient received an educational booklet, a home diary (to track their exercises), a resistance band, a pulse oximeter, and a pen drive with exercise videos. The first review was centre-based – the physician assessed patients’ risk, prescribed the appropriate home-PR programme, established patient goals, provided the PR educational booklet, and assessed inhaler technique. The therapist then demonstrated and trained the patient on the prescribed exercises, educating him/her on using the equipment and home diary. Patients were instructed to perform the self-supervised exercise at least five days per week for eight weeks (total of 40 sessions). The therapist and physician voice called the patient on a weekly basis to check in on his/her progress, including symptoms, adherence to therapy, and problems such as exacerbation, acute infection, or any difficulty encountered undertaking the exercise programme.

### Quantitative outcomes

The primary outcomes for assessing the feasibility of the home-PR programme were uptake and completion rates. The secondary outcomes were functional exercise capacity (6-Minutes Walking Test (6MWT) and Health-Related Quality-of-Life (HRQOL) (COPD Assessment Test (CAT)) measured at the start of the programme and upon completing the PR programme (at week nine or within two weeks of the last session).

### Qualitative component

Due to the COVID-19 movement restriction orders during the study period, two rehabilitation physicians (SCC and JPE) conducted semi-structured interviews via phone by two rehabilitation physicians, either in English and Malay, depending on the interviewees’ preferences. Facilitated by an interview guide (Text S1 in the **Online Supplementary Document**), we explored of perspectives and feedback on the PR programme. The questions were informed by the constructs of the Health Belief Model (HBM) and customised by the study team.

### Data analysis

We performed quantitative analysis in SPSS, version 25.0 (IBM, Armonk, New York, USA). We used descriptive statistics to describe patients’ uptake rate (the number of participants who attended the initial/baseline assessment and at least one PR session) and completion rate (the number of patients who attended the PR discharge assessment and are regarded to have “completed” the PR programme. The PR programme is considered completed by participants who completed at least 70% of the sessions recorded in the exercise diary [15]. We presented the baseline and post- therapy 6MWT and CAT scores here, but did not perform statistical analyses, as this study was not powered to show a difference; the minimally clinical importance difference (MCID) is two points for CAT scores [16,17] and 26 minutes for 6MWT [18]. We presented categorical variables as percentages and frequencies.

Interviews were transcribed verbatim in the language used during the interview (English or Malay). Two researchers (JKS and JJN) initially analysed the de-identified transcripts using NVivo software, version 12.0 (QSR International, Burlington, Massachusetts, USA), applying inductive thematic analysis to identify recurring themes in the data. Prior to the full analyses, the researchers read the transcripts several times to become acquainted with the data. Following the first round of coding, discussed and refined the initial codes with two other two researchers (JPE and SCC), before completing coding and analysis. Analysis was performed in the original transcript’s language; quotes in Malay used in article were translated into English.

### Ethical approval

We obtained ethical approval from the National Medical Research Ethics Committee, Ministry of Health, Malaysia (NMRR-20-2602-54702) and University Malaya Medical Centre Ethics Committee (2020316-8383). The Academic and Clinical Central Office for Research and Development (ACCORD) provided governance approval. All participants provided written informed consent.

## RESULTS

We recruited 30 patients (female: n = 12 (40%)) with a mean age of 59.5 years (standard deviation (SD) = 0.5) (**Table 1**). The most common diagnosis was bronchiectasis in 17 (56.7%) patients, followed by COPD in seven and ILD in six patients. Ten patients (33.3%) had been hospitalised due to a respiratory-related problem in the previous 12 months. Commonly reported symptoms included sputum production (90%), cough (73.3%), and shortness of breath (40%).

**Table 1:** Demographic profile of participants (n = 30)

Site frequency*	
University Malaya Medical Centre	15 (50)
Serdang Hospital	15 (50)
**Age in years, mean (SD)**	
Range 29-87	59.5 (0.5)
Gender*	
Male	18 (60)
Female	12 (40)
Ethnicity*	
Malay	16 (53.3)
Chinese	9 (30.0)
Indian	4 (13.3)
Bangladeshi	1 (3.3)
Diagnosis*	
COPD	7 (23.3)
Bronchiectasis	17 (56.7)
Interstitial lung disease	6 (20.0)
Level of education*	
No formal education	2 (6.7)
Primary	4 (13.3)
Secondary	11 (36.7)
Tertiary	13 (43.3)
Hospitalisation in the past 12 mo due to respiratory causes*	
Yes	10 (33.3)
No	20 (66.7)
Smoking status*	
Current smoker	0
Past smoker	9 (30)
Never smoked	21 (70)
Symptoms*	
Shortness of breath	12 (40.0)
Sputum	27 (90.0)
Cough	22 (73.3)
Others	
*Lethargy*	5 (16.7)
*Painful knees*	1 (3.3)

SD – standard deviation, COPD – chronic obstructive pulmonary disease

*Data presented as n (%) unless otherwise specified.

### Uptake and completion rate

The uptake rate for the programme was 93.3% (n = 28), with two withdrawals due to hospitalisation. The completion rate was 36.7%; only 11 patients attended the post-PR assessment in the hospital at nine weeks. Seventeen of them did not show up for the post-PR because COVID-19 restrictions prohibited travelling in Malaysia at that time.

### Functional exercise capacity and Health-related quality of life outcome measures

**Figure 1:**
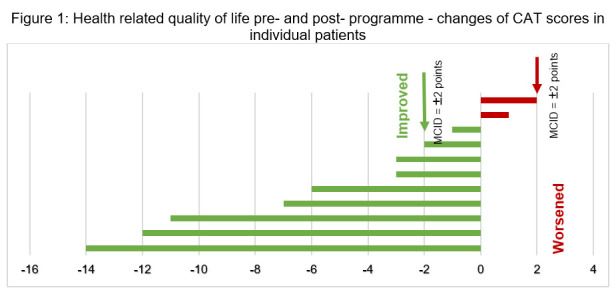
Health-related quality of life pre- and post-programme – changes of CAT scores in individual patients.

**Figure 2:**
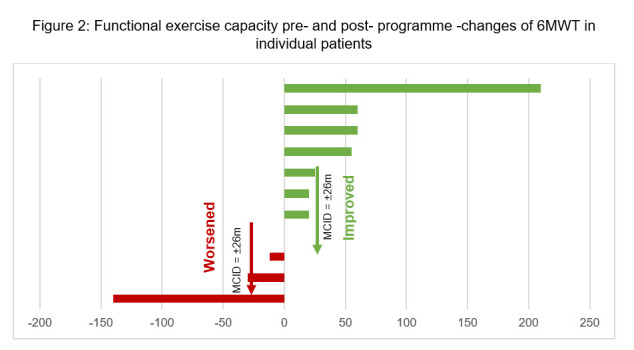
Functional exercise capacity pre- and post-programme – changes of 6MWT in individual patients.

Regarding HRQoL scores, seven out of 11 patients showed improvements, exceeding the MCID of two points for CAT scores, two showed improvements less than the MCID, and two deteriorated, but none beyond the MCID threshold. Concerning functional exercise capacity, four out of the 11 patients demonstrated improvements greater than the MCID of 26 minutes for 6MWT, five had changes that were either unchanged or less than the MCID, and two experienced worsened 6MWT post-programme (**Figure** **1** and **Figure 2**).

### Adverse events

No adverse events were reported during the home-PR programme.

### Qualitative themes

Four themes emerged from the inductive analysis: involvement of family and caregivers, barriers to home-PR programme, interactions with peers and health care professionals, and programme enhancement.

#### Involvement of family and caregivers

Family and caregivers should be given attention, as they could play an active role in implementing a home-PR programme. This is particularly essential for frail or elderly patients who require assistance completing the exercises. Caregivers should be educated on home exercises as well.

So, uh, when it comes to like physio exercises like this, right? Um, instead of just focusing on the patient, um-um, give time for the caregiver also to pick up something. So that-that means like in this case, right, like I say somewhere in the middle, don’t just focus on the patient but also on the caregiver. So, the caregiver also understands and so that they can do it at home. *– P3, caregiver.*

Patients themselves were motivated to have their families join them in the exercise. Some patients refused to participate in the home-PR programme if caregivers could not supervise them due to their work commitments.

…they need to work…They have no time to supervise the patient actually. That’s why some of the patients, they refuse to participate in this programme. *– P5, healthcare worker.*

#### Barriers to the home-PR programme

Some HCPs were concerned about patient safety because they could not monitor the patients’ exercise performance and level of exercise at home. Similarly, the patient was unsure about the extent they should exercise at home without being monitored, although HCPs could conduct phone consultation sessions in which physiotherapists could counsel and support patients individually or in groups. Safety is dependent on an accurate initial assessment and training session. The intensity of exercise is very important for the effectiveness of PR and can be successfully achieved by the patient using the 'Talk-Test'; it also guides the patient to ensure safety.

Oh, if it's at the house, we can't monitor if the patient is doing or not doing [exercises], we want to see his level, another thing is safety for him, we're worried about whether he's doing it excessively or what. *– P12, healthcare worker.*

If the situation allows it, for example, Covid has slowed down, and my movement is unlimited, I really want to go to class again. Because that is what I like, when we go to class, they [physiotherapist] will monitor us, because before that I didn't know what my oxygen level was like. So, I just did [exercises], I forced myself. But the coach [physiotherapist] said, don't force yourself. If you cannot do it, don't do it. *– P10, patient.*

“If it's at home, he [patient] seems to rarely do it [exercises] except for patients who are really passionate about it, only they will do it.” (P9, health care worker)

“Maybe later we do it for a week, uh we do something like Zoom once with the therapist so we can interact better. *– P6, healthcare worker.*

#### Interactions with peers and health care professionals

Some HCPs believed that patients might prefer the interactivity of Centre-based PR; additionally, patients can share their experiences, compare their performance to others, and develop confidence.

Because it's different near home and here. Here at the centre, he has friends. He has other friends, around 4 people. Over here we do - I do sports. Because I feel that when sports are involved, patients are more interested. They love it. What sport shall we do today, badminton... okay. They like it. *– P9, health careworker.*

Group telerehabilitation activities can be carried out to encourage exercise among peers and foster a sense of community.

The problem is to have to call one by one. Or we have a Zoom call at the same time. Which is appropriate, if it's like that at the same time, we make the Zoom, it's like we told him to share steps and circuit training. First, for breathing exercise, second, we monitor his walking exercise. *– P12, healthcare worker.*

#### Programme enhancement

The materials were printed in English and Malay languages; however, to accommodate patients of various races and backgrounds, they should also be published in Chinese and Tamil. The education materials provided to patients were reported to have inconsistencies between the Malay and English languages. This was complicated further by unclear instructions, which were visible in the differences between the text and the pictures. Consequently, patients had difficulty following exercise instructions. Patients reported that the video, which was provided in addition to the booklet, was unclear.

Oh, that video. For me, at first, I found it a little difficult to understand. But when I watched again and again, then I can understand. *– P10, patient.*

The pre-programme education session was completed in a short time and may not have been adequate to ensure that the patient understood and could perform the exercises in the book. Patientsd believe that more exercise options should be made available to them. Qigong exercises have been proposed as a complementary exercise component to promote relaxation, reduce anxiety, and alleviate clinical symptoms such as shortness of breath.

The usual reason is that when a patient comes to me, there isn't enough oxygen – it is around 92% like that, adding to that by wearing a mask, they just arrive out of breath. We tell them to calm down, we tell them to inhale for 4 seconds, hold for 4 seconds, exhale for 4 seconds. Apparently, there is- there is a change with oxygen increasing to 97%. *– P9, healthcare worker.*

The exercise programme should be tailored and personalised to patients’ ability and their own goals.

That’s one, uh, but second also I realised that over the 8 weeks and all, that if I just follow exactly what is asked of me, I wasn’t doing very much. For example, they said repeat it three to five times. So, if I just strictly follow the requirements uh, you know, uh, it may not be – I may not be adding value over the weeks. *– P2, patient*

Yes, so they should be asked what they are - what are they trying to achieve within the next week, within the next two weeks you know, something short term rather than three months from now they will forget it already. Yeah, so something short term where-where they can see when they can see it when they achieve or if they didn’t achieve, they have to set a revised goal for the next week, for example. Something that they want, they personal want. *– P2, patient.*

I also, you know, when start walking that time ah, I choose to go one round or half a round you know. Yes. You start increasing, increasing from 5 minutes until exercise—is a complete exercise for about 45 minutes, I can. You have to gradually increase the exercise, but you cannot set the target too high for them. *– P1, patient.*

## DISCUSSION

### Summary of key findings

We have demonstrated the feasibility of delivering a home-PR programme in Malaysia. This is supported by the high uptake rate (93.9%) and completion rate (36.7%) in the PR programme. However, the follow-up assessment upon completion of the PR programme was low due to the movement restrictions imposed during the COVID-19 pandemic. No adverse events were observed during the study. From the qualitative assessment, we found that family and caregiver played an active role in implementing the home-PR programme. Patient safety issues and proper guidance need to be addressed to ensure the favourable outcome of the programme.

### Comparison with other works of literature

#### Overcoming poor uptake and completion rates

Despite its benefits, the global uptake and completion rate to PR programmes remains relatively low; almost 40% of the patients did not complete the programme [19,20] which is consistent with our findings. Most studies only included patients who attended at least 70% of the therapy session. In our case, the movement-controlled order as a result of the pandemic during the study maybe one of the main factors lead to poor attendance of the post PR follow-up assessment upon completion of the PR programme. Poor transportation, inconvenient timing, and disruption to daily routines were consistently identified as barriers to both participation in and completion of the PR programme [19]. Patients with CRDs frequently experience disabling symptoms such as shortness of breath and reduced effort tolerance, making traveling to the hospital more challenging. Furthermore, due to the risk of contracting COVID-19 infection, most patients with CRDs prefered to minimise their hospital visits during the pandemic. The home-PR programme is an alternative model that could overcome these barriers, and can also improve uptake, as sessions can be delivered remotely and patients can complete them at their convenience.

#### Adapting to remote delivery in Malaysia

Over 85% of centres worldwide use a centre-based programme, with only less than 5% offering a home-PR programme [21]. First implemented in 1994, its effectiveness was comparable to that of a hospital-based PR programme [22]. The COVID-19 pandemic has resulted in significant changes in the delivery of PR. These include more teleconsultation, telemedicine, virtual exercise training, and home-PR programmes [23]. We developed and implemented the home-PR quickly in response to the pandemic, though discussions with RESPIRE colleagues undertaking similar projected helped resolve issues. The development of this home-PR programme proved beneficial for the COVID-19 period. In general, the home-PR programme promotes more frequent exercise sessions, which are often supplemented using diaries and telephone calls. Here we used simple equipment such as a resistance band, oximeter, educational booklet, and diary to ensure patient accessibility and acceptability. Patients performed upper limb resistance training using a resistance band and lower limb endurance exercise by walking as opposed to expensive stationary bicycle. Many of the PR programmes were adapted from various guidelines from high-income countries, which employ a variety of components and methods for programme delivery and monitoring and may not be deliverable in the same format in our local setting due to differences in resources, healthcare settings, cultural backgrounds, and disease spectrum. We opted for unsupervised exercise as opposed to supervised exercise as there is inadequate infrastructure, facilities, and hardware to enable the latter. We could not develop applications and provide remote support in such a short period of time and during pandemic when health care resources were limited. Individuals who want to develop home-PR programmes should first understand their local context and adapt their home programme accordingly to avoid frustration and failure.

#### Remote assessment

6MWT is a common outcome measure used in centre-based PR. It cannot be conducted at home because there is rarely enough space for the required 30-metre track [24]. The modified incremental step test may be a viable option for the home environment. It was shown as reliable and responsive in assessing exercise capacity in patients with CRDs who attend PR [25] and could thus be implemented in the home-PR programme as an alternative way to assess exercise capacity at home and aid home exercise prescription for progression in home exercise prescription. These findings imply that an effective rehabilitation dose can be delivered via phone calls with minimal resources and supervision.

#### The role of family

We found that family members and caregivers are encouraged to play an active role in the home-PR programme. This is supported by some studies which have shown the importance of providing physical and emotional support to patients with CRDs, which may lead to a higher completion rate [26-28]. One study that included family members in PR programme concluded that this could enhance the family’s skills in managing CRDs [29]. Some considerations for the home-PR programme include proper supervision from the HCPs and interactions with peers. Peer support is a vital component in the care of patients with CRD and engaging them in supporting the provision of PR by increasing uptake and compliance to the intervention [30].

#### Clinical practice implications and future research

We developed a home-PR programme for patients with CRDs in a short period with limited resources. The plan for the improvement of home-PR program will be to strengthen the education and exercise components. In our resource-constrained setting, we were unable to deliver a formal comprehensive PR programme to patients with CRDs nationwide. As we were the first in the country to implement a home-PR programme, we conducted a teach-the-teacher workshop on a home-PR programme to foster nation-wide scale up and extend its benefits to more patients with CRDs. We managed to identify some challenges faced by HCP while planning to implement the PR programme. We have also conducted dissemination meetings with stakeholders (e.g. policy makers) to ensure that we could implement a large-scale intervention in the future. This study provides useful information for future full-scale studies on implementation of PR in Malaysian health care facilities.

### Strengths and limitations of the study

As this is a feasibility study, we did not calculate a sample size, so we selected 30 patients to assess the feasibility of delivering the home-PR and to inform a larger trial, with consideration to resource constraints. A fully powered multi-centre randomised controlled trial would be needed study to determine the effectiveness of home-PR in the community. Thus, we intended to perform a three-arm, individually randomised, assessor-blinded hybrid-1 implementation trial to evaluate the clinical effectiveness of PR in patients with CRD. We only looked at the short-term effects of PR; however, future studies should examine its sustainability. The major problem we encountered during the post-PR assessment was that most of the patients (n = 17) could not to attend the assessment at the centre due to COVID-19-related movement restrictions regulation; thus, we were unable to assess their exercise capacity. We interviewed only four patients, four caregivers, and four health care professionals, so their views cannot be generalised to the national population. However, two rehabilitation physicians conducted the interviews and discussed their findings with the wider group to ensure a balanced interpretation.

## CONCLUSIONS

home-PR is feasible and may help address a gap in service delivery during the pandemic and enable socially distanced delivery of the programme. The existing programme can be further improved to maximise the benefit obtained. We hope that this study will pave the way for large-scale research studies and the routine implementation of PR in Malaysian health care facilities in the future.

## Additional material


Online Supplementary Document
