# Supplementary material for: Developing a home-based pulmonary rehabilitation programme for patients with chronic respiratory diseases in Malaysia: A mixed-method feasibility study
Source: J Glob Health. 2023 Oct 27;13:04099. doi: 10.7189/jogh.13.04099 (PMC10602205; doi:10.7189/jogh.13.04099)
Supplement: Online Supplementary Document [file jogh-13-04099-s001.pdf]

Qualitative Topic Guide for Health Care Professionals:

1. Let's start with the pulmonary rehabilitation (PR) programme that you have conducted recently.
   (a) Can you describe the home-based PR programme?
   (b) What do you think of this home-based PR programme?
   (c) Do you think all patients with chronic lung disease should join this programme? (Reasons for yes or no response)
   (d) Do you see any changes in the patients after they join this programme? What are the changes? (If no change, why do you think there is no change?) Beneficial to patients or not? (Reasons)

2. Tell me what are your experiences conducting this programme.
   (a) How does this programme differ from your existing programme?
   (b) What are the benefits of the home-based PR programme?
   (c) What are the challenges you faced while conducting the home- based PR programme?
   (d) Are you able to complete conducting the programme within the scheduled time frame?
      - Probe barriers
      - Probe facilitators
   (c) What do you think about the education/ video material/ assessment tools/ exercise/ chest physiotherapy given during the session? Are there anything that you did not like?
      - Probe likes/dislikes about each component
   (d) Do you find home- based PR programme is something acceptable to patients? Are they willing to participate?
   (e) Can you tell me if this programme changes your management?
      - Probe in what aspect

3. Can you tell how we can improve this programme?
   Probe on:
   (a) How to make it easier to follow/ attend

4. I am about to end this interview. Do you have any other comments or suggestions?

Qualitative Topic Guide for Patient and Family Members:

1. Let's start with the home-based pulmonary rehabilitation (PR) programme that you joined recently.
    - (a) Can you tell me briefly what do you think about the home-based PR programme? Prompt: easy/difficult to follow; (with reasons)
    - (b) Why do you think you are asked to join this programme?

2. Tell me what are your experiences going through/ not completing this programme?
    - (a) What are the main challenges you faced while going through the programme?
    - (b) Do you have any problem handling the equipment or any technical issues? (the use of pulse oximeter, pedometer, loading video, smart phone)
    - (c) Do you have any problem in recording the home diary?
    - (d) Are you able to complete the programme?
        - Probe barriers (prompt: timing of the session)
        - Probe facilitators
        - Reasons for not compliant to home-based programme (if relevant, prompt: lack of time, competing activities, other medical conditions that limit ability to take part)
    - (e) What do you think about the education/ video material exercise/ chest physiotherapy/ home diary given during the session? what you did not like about the system?
        - Probe likes/dislikes about each component
    - (f) Do you notice any change in your chest condition after the programme?
        - Probe if he/she gets better or worse & in what aspect

3. How we can improve this programme?
    - (a) Make it easier to follow

4. I am about to end the interview. Do you have any other comments or issues that you would like to bring up?
